# Crystal structures of the carboxyl cGMP binding domain of p*lasmodium falciparum* cGMP-dependent protein kinase reveals a novel salt bridge crucial for activation

**DOI:** 10.1186/2050-6511-14-S1-P33

**Published:** 2013-08-29

**Authors:** Jeong Joo Kim, Eduardo Sanabria Figueroa, Eugen Franz, Daniela Bertinetti, Friedrich Herberg, Choel Kim

**Affiliations:** 1 Department of Pharmacology, Baylor College of Medicine, One Baylor Plaza, Houston, Texas, USA; 2 Department of Biochemistry, University of Kassel, Kassel, Germany; 3 Verna and Marrs McLean Department of Biochemistry and Molecular Biology, Baylor College of Medicine, Houston, Texas, USA; 4 Current address, Department of Pharmacology, School of Medicine, Emory University. Atlanta, GA, USA

## Background

*Plasmodium falciparum* cGMP-dependent protein kinase (pfPKG) is a validated therapeutic target of malaria. As a key regulator of its life cycle, pfPKG plays a crucial role in both the sexual and asexual blood-stages that cause malaria pathology. Inhibiting pfPKG blocks proliferation and transmission of the parasite [1,2]. However the development of pfPKG-specific inhibitor has been greatly hampered by the lack of high-resolution structure information to guide drug design.

Targeting the ATP binding site of pfPKG is an approach commonly associated with low specificity and toxicity [3]. Therefore, we aim to target a domain that is unique to this kinase, the cyclic nucleotide binding (CNB) domain. Since previous studies demonstrated the fourth-cyclic nucleotide binding (CNB-D) domain of pfPKG to be the most important for the kinase activation [4] we focused on this domain to understand its role in cGMP dependent activation.

## Results

To understand the functional roles of the CNB-D domain in pfPKG activation, we determined crystal structures of CNB-D with and without cGMP at 1.9 and 2.0 Å, respectively. The structure of the cGMP complex reveals that CNB-D binds cGMP through [1] leucine and arginine residues on the β5 strand and a threonine within the phosphate binding cassette (PBC) that interact specifically with the guanine moiety through hydrogen bonding [2] a conserved arginine residue within the PBC that provides a hydrophobic capping interaction with cGMP. The structure also shows that the side chain of the conserved arginine residue within the PBC interacts with two conserved glutamine and aspartic acid residues on the αC-helix forming a stable salt bridge. Mutation of the salt bridge forming residues drastically reduces its affinity for cGMP and increases activation constants (*Ka*) of the full-length kinase. Furthermore, comparison with the apo structure shows that the salt bridge stabilizes the cGMP bound conformation and shields the cGMP binding pocket from solvent by anchoring the highly dynamic αC-helix.

## Conclusion

Our structures and accompanying mutagenesis studies demonstrate functional roles of the CNB-D domain in cGMP binding and activation. Our crystal structures also reveal the molecular details of cGMP pocket and the unique salt bridge that can be differentially targeted for the development of pfPKG-specific inhibitor.
